# Analyzing Factors Influencing Situation Awareness in Autonomous Vehicles—A Survey

**DOI:** 10.3390/s23084075

**Published:** 2023-04-18

**Authors:** Henry Alexander Ignatious, Hesham El-Sayed, Manzoor Ahmed Khan, Bassem Mahmoud Mokhtar

**Affiliations:** 1 College of Information Technology, United Arab Emirates University, Al Ain P.O. Box 15551, United Arab Emirates; 2 Emirates Center for Mobility Research, United Arab Emirates University, Al Ain P.O. Box 15551, United Arab Emirates; 3 School of Electronics, Communications and Computer Engineering, Egypt-Japan University of Science and Technology, Alexandria 21934, Egypt

**Keywords:** automated driving system (ADS), autonomous vehicle (AV), data pre-processing, multimodal fusion, situation awareness (SA), rule framing, machine learning (ML)

## Abstract

Autonomous driving of higher automation levels asks for optimal execution of critical maneuvers in all environments. A crucial prerequisite for such optimal decision-making instances is accurate situation awareness of automated and connected vehicles. For this, vehicles rely on the sensory data captured from onboard sensors and information collected through V2X communication. The classical onboard sensors exhibit different capabilities and hence a heterogeneous set of sensors is required to create better situation awareness. Fusion of the sensory data from such a set of heterogeneous sensors poses critical challenges when it comes to creating an accurate environment context for effective decision-making in AVs. Hence this exclusive survey analyses the influence of mandatory factors like data pre-processing preferably data fusion along with situation awareness toward effective decision-making in the AVs. A wide range of recent and related articles are analyzed from various perceptive, to pick the major hiccups, which can be further addressed to focus on the goals of higher automation levels. A section of the solution sketch is provided that directs the readers to the potential research directions for achieving accurate contextual awareness. To the best of our knowledge, this survey is uniquely positioned for its scope, taxonomy, and future directions.

## 1. Introduction

Autonomous vehicles can sense their environment and take the appropriate instant decisions to react to environmental events. Autonomous cars use a wide range of sensors such as radar, LiDAR, sonar, GPS, odometer, and inertial measurement units to capture their environmental information. The following facts explain some of the interesting outcomes of autonomous driving. Autonomous vehicle companies with a valid permit along with a safety driver in California reported that their vehicles drove nearly 2.9 million miles during the most recent period time (2019–2020). Reports provided by Aptiv and Lyft in Las Vegas in February 2020, state that one lakh riders in Rob-Taxi-Drives have given a high rating regarding the safety of the travel [1]. Surveys conducted by Renault-Nissan-Mitsubishi reveal that 55% of small fleet owners have assured to convert their fleets to be fully autonomous within 20 years [2]. Nearly 54% of respondents to a recent Northeastern University/Gallup survey has reported that they are unlikely to use fully self-driving cars when they arrive on the roads [3]. In current trends, vehicle manufacturers, fleet management, and the public are more fascinated with using autonomous vehicles (AVs).

Human errors cause 94% of road accidents according to a recent technical analysis by the National Highway Traffic Safety Administration (NHTSA) [4]. In this context, Automated Driving Systems (ADSs) are being developed with the goal of decreasing accidents, lowering emissions, transferring the mobility-impaired, and reducing the stress associated with driving [5]. The yearly societal benefits of ADSs, if widely adopted, are expected to reach about USD 800 billion by 2050 as a result of reduced traffic congestion, reduced road fatalities, reduced energy consumption, and enhanced productivity due to the reallocation of driving time. The availability of new sensors types such as LiDAR, radars, Velodyne’s, and catalyzed ADSs, integrated with advanced ML techniques such as deep learning and innovative technologies such as computer vision, improves the accuracy of the perceived vehicular environment information (SA), for effective decision-making. Furthermore, the creation of ADSs with varying degrees of automation was prompted by a rise in the public interest and business potential. Robust automated driving in urban contexts, on the other hand, is yet to be achieved [5].

Accidents induced by immature systems [6] erode trust and, in some cases, result in casualty. As a result, a thorough examination of unsolved challenges and current state-of-the-art is judged necessary in this case. Autonomous vehicle technology has many advantages over manual driving. Some of the key advantages are reduced accidents, minimized traffic congestion, better pollution control, increased lane capacity, and less fuel consumption, which in turn reduces the overall cost of commodities and transportation. The perception systems must be accurate in giving a precise understanding of the environment. They must be able to function properly in inclement weather and even when certain sensors are damaged or faulty. Sensor systems must be complex and effective in order to collect environmental data as well as data related to autonomous vehicle characteristics. The vehicular environment data collected from several devices such as sensors, thermal cameras, radars, etc., exhibit heterogeneous multimodal characteristics, which further complicates the processing tasks of decision-making in the AVs [7]. Instant decision-making for critical maneuvers is an important task to ensure reliability and safety, which includes lane changing, platooning activities, rerouting, braking, overtaking, etc. For effective decision-making, more clarity is needed in the data collected from different sources to frame intelligent rules for decision-making. Since different sensors capture and store vehicular data that exhibit multimodal characteristics, further processing of the data to frame decision rules becomes a challenging task. Hence, effective pre-processing tasks namely data-cleaning and fusion of the multimodal data into a unique format, which improves the SA of the vehicular environment and facilitates the rule framing mechanism for effective decision-making is an imperative task for autonomous driving [8,9].

AD will not be widely used anytime soon due to various challenges such as the cost associated with technology, safety and security issues, legalization problems, and reduced job opportunities. To some extent, however, it is still feasible to predict its prospective impact and benefits. ADSs are widely used today in many developed countries due to the prime advantages listed below.

- Problems that can be resolved include the following: decreasing pollutants, preventing road accidents, and reducing traffic congestion;
- Possibilities emerge, such as reallocating driving time and conveying the mobility handicapped;
- New trends include mobility as a service (MaaS) consumption and the logistics revolution.

The widespread use of AD has the potential to reduce societal losses caused by erroneous human behavior such as distraction, drunk driving, and speeding [10]. The older generation (those over 60 years old) is growing at a greater rate than the younger generations [11]. Using ADSs to increase the mobility of elderly people can have a significant impact on the quality of life and productivity of a large segment of the population. A new trend is moving away from owning a personal automobile and toward using Mobility as a Service (MaaS). Currently, ride-sharing is less expensive than owning a vehicle with an annual mileage of fewer than 1000 kilometers. By 2030, the proportion of owned versus shared automobiles is predicted to be 50:50 [12]. This trend could be accelerated by a large-scale deployment of ADSs. In this paper, we survey recent advances in multimodal fusion followed by SA, and instant decision-making strategies in autonomous driving. We also discuss the challenges and research gaps in existing research.

The paper is organized as follows. Section 2 presents the background concepts related to this survey, Section 3 discusses the major research contributions in the literature related to the mandatory areas of autonomous driving, namely multimodal fusion, situation awareness, and decision-making. The section also highlights research limitations and identifies the research gaps in the existing literature. Section 4 discusses the identified existing gaps in the literature and the next future steps in the research, followed by the conclusions in Section 5.

## 2. Roadmap of the Survey

The goal of fully autonomous vehicles is still a long way off, and it could take a few more years. Many activities around the world are leading to and evaluating vehicle autonomy in various environments, such as equipping vehicles with technologies and implementing Level 3 and higher use cases on various pilot sites, such as fully managed, partially controlled, highways, and so on. This section discusses the background information related to conceptual awareness, multimodal fusion, and the decision-making principles related to autonomous driving.

The Society of Automotive Engineers states that in order for fully automated vehicles to achieve Level 5 autonomy, they must overcome the difficulties associated with teaching them to drive more safely than people (SAE). This inhibits and increases the cost of implementing fully automated vehicles. Radar, LIDAR, computer vision, sonar, and GPS are some of the sensors used by autonomous vehicles to perceive their environments. To calculate navigational directions, avoid obstructions, and read suitable indicators such as road signs, these perceiving devices process the acquired sensory information. Autonomous vehicle research teams gather test driving information from a large number of hours in various areas across the globe. To train algorithms for effective decision-making in AVs, this tremendous volume of data must be collected, distributed, processed, and analyzed. The main difficulty is figuring out how to efficiently manage all of the test-generated data and instruct the vehicles to make decisions more quickly in a range of circumstances. Creating control systems that can effectively navigate roadways and comprehend information requires teams working toward SAE Level 5 autonomy to gather, store, analyze, and interpret enormous amounts of sensory data. In order to drive correctly and safely, contemporary AV technology uses advanced computations to aggregate data from multiple sensing devices and other sources. Building vehicle autonomy with machine learning and artificial intelligence (AI) involves ongoing implementation and developing expertise. The speed at which autonomous vehicles are developed is determined by research and development as well as the technological ability to capture, store, and evaluate huge volumes of sensory information in order to not only translate data into advanced computations that enhance AD precision and efficiency but also to develop smart autonomy. Auto manufacturers who can speed up this phase will have a leg up on the competition for SAE Level 5 autonomy [2,7,13].

**Figure 1:**
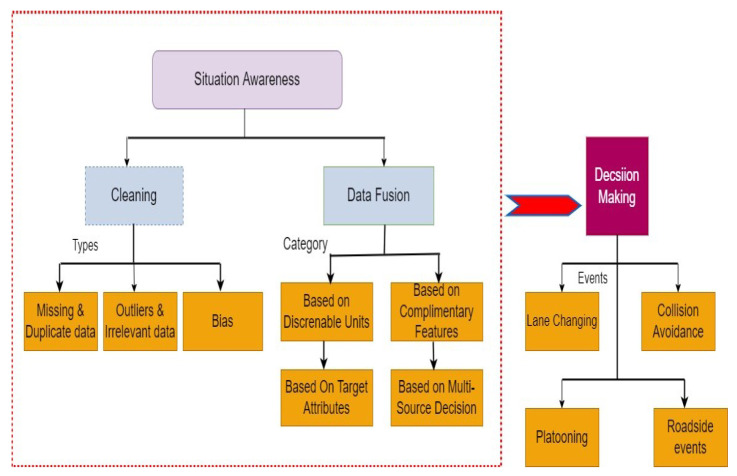
Overall flow of the survey.

Figure 1 illustrates the architecture according to which the survey is organized. Since more contributions have been done to the data cleaning process, in the first phase, more priority is given to the multimodal fusion task, which plays a vital role in improving the SA of the AVs. In data fusion, various articles are grouped based on discernible units, complimentary features, target attributes, and multi-source destinations. The outcome of the first phase leads to effective decision-making tasks in AVs. Various contributions from different authors related to decision-making in AVs are analyzed based on various events, namely lane changing, collision avoidance, various roadside events, and platooning. The outcome of the analysis is identifying the pitfalls associated with the articles in the first phase, which degrades the SA and impacts the accurate decision-making in the AVs.

The following sections discuss the mandatory information related to the key factors, namely situation awareness, multimodal fusion, and decision-making in AVs.

### 2.1. A Brief Overview about Situation Awareness

According to Ref. [14], SA is defined as “the perception of the elements in the environment within a volume of time and space, the comprehension of their meaning and the projection of their status in the near future”. SA bridges the gap between what we know about the environment, what is occurring in it, and what could happen in the future. Endsley split the processes of SA into three levels of responsiveness: Level 1, ‘Perception’, is the fundamental level of awareness that encompasses the perception of environmental signals. Level 2, ‘Comprehension’, necessitates an analysis of the current situation, taking into consideration many fragments of data and their comparative relevance to one another in order to understand what we are perceiving. Level 3, ‘Projection’, is the capacity of the operator to make predictions about the future condition of things in their surroundings. It is a serial product of Levels 1 and 2 in their surroundings [15,16].

The three degrees of SA correspond to how an autonomous vehicle perceives and analyses the driving environmental scenarios. In AVs, the automatic process ‘perceives’ using sensing devices such as LIDAR, radar, and numerous cameras that can perceive information ‘through’ walls and beneath the road surface, but is confined to visual and audio inputs [17]. The AV sensors, on the other hand, might be deceived, and false positives can result in emergency braking maneuvers. For instance, in the first step of the Move-UK project, the AV misinterpreted a cloud of exhaust smoke circling over the roadside for a fixed material and ordered the vehicle to stop, illustrating that human interference may be necessary even at the level of basic perceptional decisions since they may call for some level of coordinated comprehension. In terms of Level 2, ‘Comprehension’, AVs presently lack the AI required to attain human-like comprehensive insight. Many edge situations are context-dependent, and an AV may miss essential or irrelevant facts that a person would notice [18]. In Level 3, ‘Projection’, AVs are currently unable to make accurate forecasts since real-world driving is unpredictable and necessitates both proactive and reactive judgments to avoid dangerous scenarios from arising [19].

**Figure 2:**
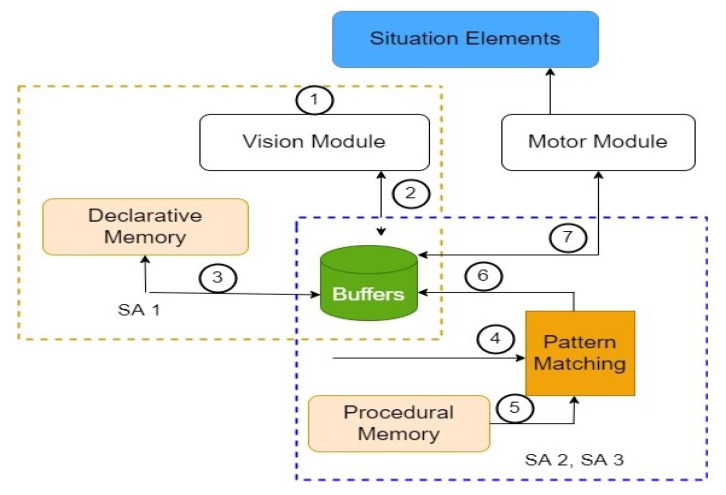
Overall architecture of the situation awareness in the AVs.

Figure 2 explains the overall architecture of Situation Awareness in AVs. The role of every interconnected module that contributes towards SA is explained in the following paragraphs. The first module is the vision module, which selects and registers certain SEs into their short-term sensory store after successful filtering is done using selective attention methodology. The buffers access the vision module’s environmental data and choose the proper action condition from declarative memory. Once the (if) part of the procedural memory (which contains the rules) matches with the rules stored in the buffer, the corresponding (then) part in terms of the actions is fetched from the motor module using a pattern-matching algorithm. The numbers highlighted in Figure 2, depict the hierarchy of execution of various tasks related to SA.

Many eminent researchers have contributed to the importance of situation awareness in AVs. Useful surveys and articles covering various aspects of SA such as different methods used to record situation awareness, various measurements used to compute the SA, and important SA architectures that are used by the manufacturers and researchers to organize the procedures by collecting the environmental data for effective decision-making in AVs have been published to help the research community acquire in-depth knowledge of the field of AVs.

### 2.2. Basics of Data Fusion and the Perception System of AV

**Figure 3:**
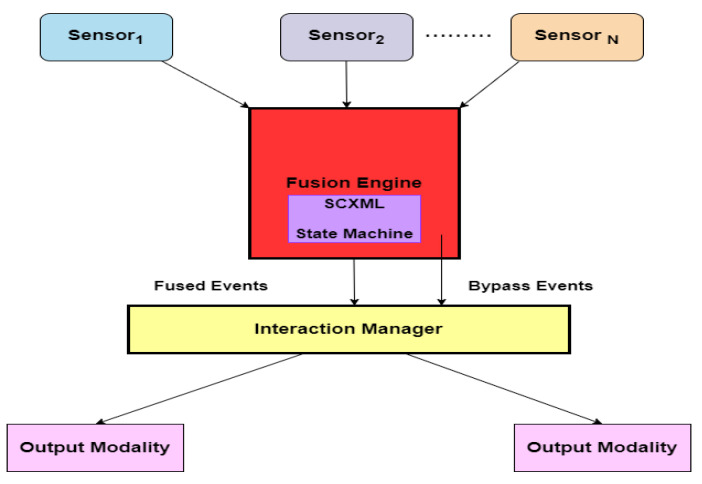
Multimodal fusion architecture.

This paragraph explains the key concepts related to multimodal fusion. The main focus of this section is to highlight the readers regarding the importance of data fusion towards SA of the AVs. Different sensors are deployed in autonomous vehicles to gather information related to the vehicular environment. Normally, sensor data is heterogeneous and represents multimodal characteristics. Processing multimodal data is difficult and henceforth they must be fused and converted into a unique format, which facilitates further processing. However, fusing multimodal data is a complicated task and requires more mathematical transformations and calculations. Various eminent articles related to fusing heterogeneous vehicular data are analyzed and their main contributions followed by the drawbacks are discussed in this section. This section highlights the complete picture of various innovative strategies proposed by various authors to fuse and convert vehicular data into a unique format. Figure 3 illustrates the overall architecture of data fusion. Multimodal data is acquired from several sensing devices. The collected vehicular information is forwarded to the fusion module where data is fused into a single format. The fused data is passed to the interaction manager, which handles specific tasks—namely handling input and output in various modalities such as speech, video, etc. The output modality focuses on integrating multimodal data (e.g., speech and video). To carry out these transformations, advanced mathematical models are used. To date, a multimodal fusion of sensor data is a complex activity due to various reasons such as the limitations associated with the sensors, differences in temporal and spatial resolution data format, and geometric alignment. Other challenging issues such as the uncertainty of reliable data sources, inconsistent data, missing values, and heterogeneous nature make this process puzzling to many researchers and autonomous vehicle solution providers. This section discusses the contributions and methodologies proposed by many authors regarding the multimodal fusion of sensor data for autonomous driving.

In the IoT world, a massive volume of data is generated in a short period. The question of how to make such a massive volume of data exact and highly accurate remains unsolved, despite the fact that information quality plays a crucial part in decision-making. It is vital to have accurate and reliable information. This can be accomplished through data or information fusion (terms that can be used interchangeably). Because of the following reasons, data fusion plays a key role in the success of the Internet of Things (IoT) paradigm [20]:

- Data from multiple sensors and sources are combined to create something more smart, definite, intuitive, and precise information. The data from each sensor may not make much sense on its own;
- Computing the (N) independent observations yields a statistical benefit of fusion; one may expect the data to be combined in the best possible way;
- Manufacturing minimal power consumption sensors, which reduces the frequent replacement of batteries during their lifespan, is a key problem in IoT. This prevailing condition reduces the demand for energy-efficient sensors in the market. It is well known that high-precision sensors use a lot of power;
- To address this problem, a set of low-accuracy sensors with very low power consumption can be deployed. Data fusion allows for the creation of extremely accurate information;
- Data fusion can assist with IoT big data challenges because we are combining data from various sensors into more specific and reliable information;
- Another significant advantage of data fusion is that it helps in the concealment of essential data or semantics that are accountable for the fused outcomes. Military applications, some important medical sections, and intelligence buildings, are examples of this.

Based on the mathematical approaches used, data fusion strategies can be divided into three categories:

- Artificial Intelligence (AI) based approaches such as classical machine learning, fuzzy logic, artificial neural networks (ANN), and genetic assessment;
- Probability-based methods such as Bayesian analysis, statistics, and recursive operators;
- Evidence-based data fusion strategies based on theory.

#### 2.2.1. History of Fusion

With roots in the 1980s, the SDF group has been around for 40 years. In the era of data fusion, information, and decision-making systems investigate distributive fusion. This has involved groups such as Garvey at SRII, working on AI, Reid at Lockheed, working on various assertion monitoring, and Bar-shalom at Systems Control, who is developing a combined probability-based data association filter and data fusion [21]. Over the decades, information fusion has progressed in leaps and bounds. The SIAP concept created a hypothesis of a tree structure with a rule-based and signal-understanding mechanism. For instance, a layer of regulations received audio energy from hydrophones and extrapolated signaling principles were utilized to carry out the conceptual transition over different treatments to identify and locate vessels [22]. Since the 1980s, there have been ground-breaking developments in computing and networking, as well as widespread business establishments of sensor and data fusion. Over 100 businesses claim to offer AI that incorporates ML and SDF. In the 1980s, for example, researchers aimed for cognitive computation techniques to maintain autonomous land vehicles (ALV). A sensitive camera system was used in the 1985 ALV road demo to identify road borders at noon rather than shadows at night [23]. Further tests demonstrated that environmental changes, such as mud on the road, had restrictions. As a result, the AI industry (hardware and software) tanked in the 1980s. In the 1990s, the merging of AI and SDF became obvious. The use of probabilistic graphical models to enable model-based reasoning became popular, mainly due to: (1) uncertainty and inference being rigorously represented, (2) model-based learning from data, based on evidence and allowing for explainable results, and (3) logical relationship extensions. The broad discussion of AI focuses on its history, which is represented in a variety of ways. The three stages of development [24] are one example. Expert systems were the first phase, in which researchers attempted to emulate human experts in speech and signal understanding. In the second stage, probability modeling was used in descriptive statistics for object and situation identification. Advances in neural networks using DL for video tracking and natural language processing are included in the current phase, which is the third phase. The first wave of AI for model-based development with handmade (or labeled) knowledge includes both the first and second phases. The third phase encourages a second wave of AI, which involves statistical learning based on data. The third wave of contextual adaptation is being ridden by today’s AI/ML techniques. Machine learning, AI, Fuzzy Logic, Deep Learning, and data fusion are all used to gradually develop intelligence. Various works have looked into the process of developing intelligence for IoT-based smart communities. Luc Julia, a former Apple Siri director who is now the Vice President of Samsung Open Innovations, introduced the Samsung Architecture for Multimodal Interactions (SAMI), which is part of Samsung’s AI strategy for its IoT-based approach [25]. In its pursuit of IoT, Google just acquired DeepMind, an AI company. Boston Dynamics, a robotics business, and Nest Labs are two more recent large acquisitions. In the Internet of Things, artificial intelligence is increasingly widely employed for sensor fusion, event processing, and location. The major contribution of AI-based data fusion research and development is discussed in Ref. [25]. The following questions were addressed by each panel list.

1. What is the issue with SDF?
2. Which SDF problem component requires ML, and why?
3. Which scenarios effectively use AI/ML techniques to solve fusion-related problems?
4. When does it become difficult to use machine learning for SDF?

**Figure 4:**
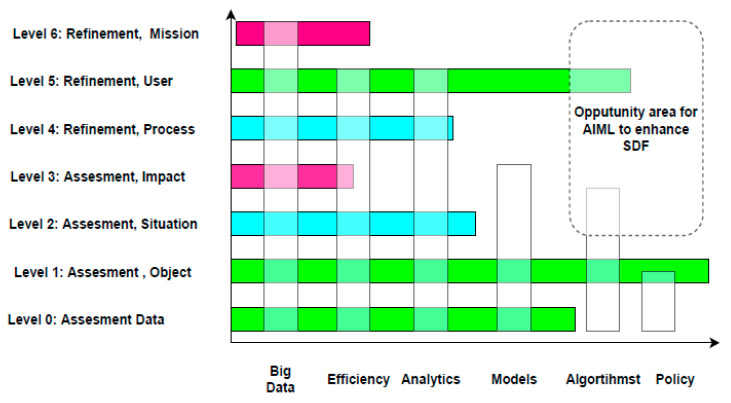
Fusion history.

While Table 1 summarizes the discussion, each panelist chose specific concerns that were relevant to their Sensor Data Fusion (SDF) and AI/ML experiences. To determine the motivation behind the panelists’ viewpoints, Figure 4 displays a broad interaction between AI/ML problems with the Data Information Fusion Group (DFIG) model levels (Level 0–6). For instance, AI/ML enables big data analysis that spans all layers of data fusion. For instance, AI/ML enables big data analysis that spans all layers of data fusion. The classic SDF levels are supported by a variety of AI/ML model creation and efficiency methodologies. There exist few AI/ML techniques, which allow for contextual information and impact analysis, nor are there many AI/ML policies that need to be defined. Similarly, several AI/ML technologies that can enhance or complement SDF user refinement are used in SDF object assessment. In Figure 4, vertical bars reflect the proportion of AI/ML society interaction, while the horizontal bars depict the degree of subject areas and/or attention given to the data fusion group. For instance, “big data” has an impact on all aspects of data fusion, whereas “policies” are being developed exclusively for object assessment, not for situation assessment. The data fusion society emphasizes the necessity for all AI/ML subjects in target evaluation, but debates on “mission management” rely on big data, landing in information management, rather than AI/ML policies for “mission management”. Finally, the green hues depict a strong overlapping between AI and SDF, whereas blue is slightly overlapped, and while red did not make any impact in the panel discussion. In order to activate the conceptual awareness and impact evaluation, as well as to process optimization, further research in AI/ML techniques is required, as shown in the top right corner of Figure 4. The rest of the article contains major points raised by the panelists.

The advancements in computer hardware over the days have improved the efficiency and accuracy of the systems to a great extent. The process of integrating information from various sensors is known as multisource and heterogeneous information fusion (MSHIF). MSHIF creates an accurate perception and detection of the vehicle’s surrounding environment by avoiding the perceptive limitations and variations caused by a single sensor. Additionally, it enhances the system’s capacity for extrasensory perception. Currently, MSHIF technology is used in multiple domains other than autonomous driving. Other intriguing areas include object tracking and identification, robotic devices, versatile devices to monitor human activities, remote sensing, human-machine interaction, simultaneous localization, and mapping. This is evidence that proves that multimodal fusion is an important task in improving the SA for effective decision-making in AVs.

#### 2.2.2. Sensors in Fusion Perception System

The ambient data that an AV needs to make wise decisions is acquired in large part through sensors. The type and performance of the sensors determines the quality of the information required for an AV. The AVs perceive the external environment data. Frequently used sensor types are radar, ultrasonic, cameras (including RGB-D, and infrared), LiDAR, and GPS/IMU. Object identification and location precision may be improved via many sensors because the detection capabilities and dependability of various sensors are constrained in various settings. The pros and cons of the aforementioned sensing devices, along with their detection range, are summarized in Table 1, which demonstrates the apparent operational differences between the various sensors. By combining data from several sensors, it will simultaneously enhance the AD vehicle’s overall perception capability to effectively ensure the driver’s safety.

#### 2.2.3. Millimeter Wave Radar

The radar first emits electromagnetic waves, then uses a receiving antenna to collect the dispersed wave of targets. A series of signal-processing operations are then carried out to obtain information on the targets. Currently, the three primary frequency bands used by MMW-Radars are 24 GHz, 60 GHz, and 77 GHz, with 77 GHz being the most common. Sixty GHz is currently only used in Japan, while 24 GHz will eventually be phased out. The 79 GHZ band radar provides better sensitivity to distance, velocity, and gradient. It has received widespread approval and may soon overtake other frequency bands as the standard for vehicle radar. The distance resolution $R_{res}$ and speed resolution $V_{res}$ are calculated using the below equations:

(1) $$R_{res}=\frac{c}{2B}$$

(2) $$\theta_{res}=\frac{\lambda }{Ndcos\theta }$$

where *C* is the speed of light, λ is the wavelength, and *B* is the bandwidth of the chip.

#### 2.2.4. Cameras

**Figure 5:**
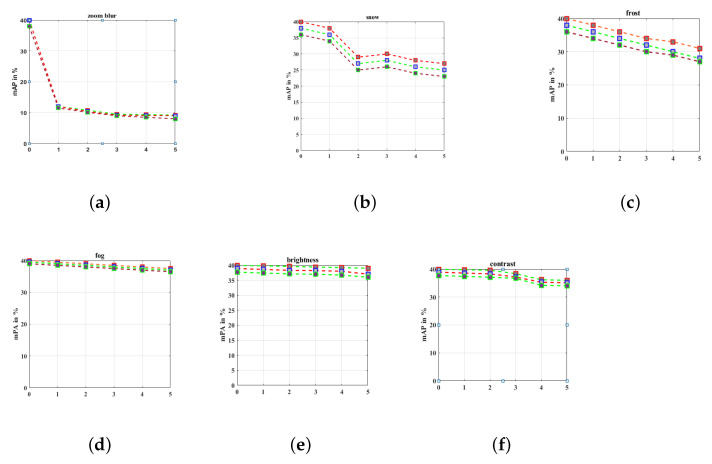
The performance of the camera influenced by different corruptions (**a**) zoom blur (**b**) snow (**c**) frost (**d**) fog (**e**) brightness (**f**) contrast.

One of the first AD system sensors is the camera, which is still the top option for producers and researchers today. Target tracking, lane detection, environment map generation, and target recognition are among the main activities the camera is used for. Deep learning (DL), which may change the conventional hand-operated feature design processes with machine learning techniques and get great interpretation ability from large data., has recently achieved good results in target detection and tracking tasks. Following the AD system’s successful completion of the object identification and object navigation duties, more conclusion responsibilities will be implemented. There are now two different types of cameras: complementary metal-oxide semiconductor (CMOS) and charge-coupled device (CCD). In addition to having a difficult manufacturing process, CCD has a greater dynamic range, lower noise, higher quantization efficiency, and prominent image characteristics in diminished light. When contrasted with CCD sensors, CMOS sensors sacrifice a few functionalities to minimize cost. Wearier differences will exist between them, and CMOS is anticipated to take the role of CCD. The authors used three datasets namely Pascal, Coco, and Cityscapes to evaluate their object detection algorithm. The results obtained are portrayed in Figure 5. The red line represents the performance of the author’s object detection model for the Coco dataset, green for Cityscape, and brown for Pascal datasets respectively representing different weather conditions. Further, from the inferences illustrated in Figure 5 it is evident that in some circumstances the perceiving precision of the cameras is diminished with a minimum score of 31.1% and a maximum score of 60.4%. Hence, it is sensible to draw the conclusion that sensors with one camera will be quite unreliable in bad weather [26].

#### 2.2.5. LiDAR

**Figure 6:**
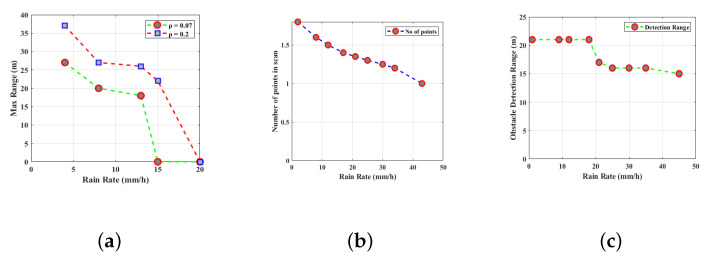
(**a**) Max range influenced by rain rate (**b**) Number of points influenced by rain rate (**c**) Obstacle detection range influenced by rain rate.

LiDAR covers 2D LiDAR and 3D LiDAR in accordance with the scanning pattern, and estimates the period to measure the distance from electroluminescent laser beams and dispersion reflected by objects. While 3D LiDAR is a multi-layer device, 2D LiDAR is a single-layer one. AD cars use 3D LiDAR more frequently, although it is more expensive. Manufacturing prices will continuously decrease as LiDAR applications and production rise, and they will eventually get to a point where most automakers can adopt them. LiDAR offers accurate and practical 3D perception abilities both during the day and at night. LiDAR is classified into three categories, including time-of-flight (TOF), triangulating LiDAR, and phase-ranging LiDAR, depending on whether motion units are present or absent [27]. TOF LiDAR in AD systems is the most common variety. According to the most recent research, LiDAR is completely capable of identifying and sensing the various mobility patterns and spatial states of pedestrians [28]. In order to detect and identify pedestrians and vehicles, the multiple-input LiDAR continuously generates a laser beam through an emitter. The transmitter then collects the destination light to create an image of a point cloud. The detailed functioning of the above-mentioned sensors is discussed in our paper [29]. Table 2 illustrates the overall summary of different sensors based on their functionality, advantages, and disadvantages. Figure 5 and Figure 6 display the findings from Ref. [30] and their analysis of how rainfall affects LiDAR. These images demonstrate how performance drops quickly when rainfall increases, including a rapid decrease in the maximal observable range, the number of markers, the extent of obstacles, and other parameters.

Figure 6, illustrates the impact of the rain rate on the performance of LiDAR sensors.

The survey addresses the research questions listed below, to highlight its main objective and guide the readers in the right direction.

1. What are the recent trends adapted to pre-process heterogeneous vehicular data?
2. What level of data accuracy is obtained by the referred studies after pre-processing the data?
3. Does the initial pre-processing tasks improve the situation awareness of the AVs?
4. Do the referred decision-making models provide instant solutions for all roadside events?
5. Which type of evaluation criteria in the referred decision-making models plays a vital role? Simulation-based (or) real-time implementation?
6. Does the survey identify the pitfalls in the existing literature?
7. What solution does this study propose for overcoming the identified drawbacks in the existing literature?

## 3. State of the Art in Data Fusion, Situation Awareness, and Decision-Making

This section summarizes the research work in the literature related to multimodal fusion, situation awareness, and decision-making related to the sensory data of autonomous vehicles. Innovative contributions and strategies proposed by eminent scholars, related to the three areas, are elaborated on in this section. At the end of each section, the summary of the referred studies is illustrated in a chronological manner, highlighting the pros and rectified cons of each referred article.

The articles related to all the areas analyzed in the survey are collected and organized based on various attributes such as pointers, methods, and sources. Pointers refer to various functionalities related to a specific area. Methods refer to different types of approaches (or) solutions used to solve the prevailing problems. If the articles directly convey the related information then they come under the category of primary source. If they indirectly refer to a focused area then they fall under the category of secondary sources. The next stage refers to the summary of the reviewed areas followed by future plans and conclusions.

### 3.1. Multimodal Fusion

Articles related to data fusion are collected based on three major divisions, namely the pointers that indicate the purpose of fusing the sensor data, different datasets, and methods that are exclusively used to fuse the complex data collected from different sensors. There are several architectural models in data fusion systems; nonetheless, applications constantly apply their own data fusion designs. The Joint Directors of Laboratories (JDL) Model, Modified Waterfall Fusion Model, Boyd Model, and Dasarathy’s functional model are some of the basic and fundamental models [31,32,33]. These models are then subdivided into layers, which are subsequently subdivided into sublayers. The heart of data fusion, however, is undeniably in the data fusion methodologies that enable ultimate fusion processing. We looked at some of the most current developments in probabilistic data fusion approaches and algorithms in this area.

The most common, easiest, and widely used methods for fusing data are probabilistic procedures. Nonetheless, they may not be as accurate as analytical methods. Probability is a major component of the underlying logic representing the majority of traditional data fusion methods [33]. However, there are some difficulties with probabilistic data fusion. According to research, probability-based information fusion systems struggle to handle assessed participation when compared to fuzzy logic. Probability-based fusion strategies have a high level of complexity when handling no monotonic logic, and cannot depict all the information required for defining and depicting sensing and data fusion operations. The Monte Carlo method, Markov Chains, and Bayesian theory have been some of the most researched and widely used data fusion techniques in recent times. Target tracking problems frequently employ probability-based data fusion methods. The Probabilistic Data Association (PDA) technique is the most well-known example of single target tracking. Several enhanced versions of PDA have been published in the literature, and they are quite effective at tracking single targets. We also noticed the situation and difficulty of multi-target tracking changes (MTT). Because track validation is challenging due to track competition, MTT uses a more efficient Joint Probabilistic Data Association (JPDA) algorithm. It employs a measurement-to-probabilities-of-association evaluation.

The Bayesian data fusion methodology is the most traditional, as it is generally acknowledged and utilized for data fusion, and it is at the heart of many data fusion systems. Based on the probability theory, it mixes multimodal data. In the Bayesian technique, priors are defined and specified, while posteriors are calculated. Refs. [34,35], have proposed several data fusion models that follow the Bayesian approach. Two approaches for dispersed target detection have been proposed in Ref. [34]: the distributed Bayesian methodology, and the Generalized Likelihood Ratio Test (GLRT) for wireless sensor networks (WSNs).

To manage objective kinetics variations in rapidly changing conditions and varying precision criteria, a blended computational solution and a responsive data fusion estimation technique are needed. Typically, the Information Matrix Filter (IMF) approach is employed to address the aforementioned issue. A hybrid tracking technique that handles entity relationships by fusing the Bayesian filter and Markov chain Monte Carlo (MCMC) sampling is put forth by a different study; Ref. [34]. A user interface known as IoT middleware unifies and streamlines the communication between various “Things” and the internet. One of the most important elements of IoT middleware is event processing. Bayesian Model Averaging (BAM) was utilized to look into predictive fusion analytics in Ref. [36]. Data fusion, which is used to achieve proactive complicated event processing, can help prediction analytics. Using this approach, large-scale IoT applications are developed.

**Figure 7:**
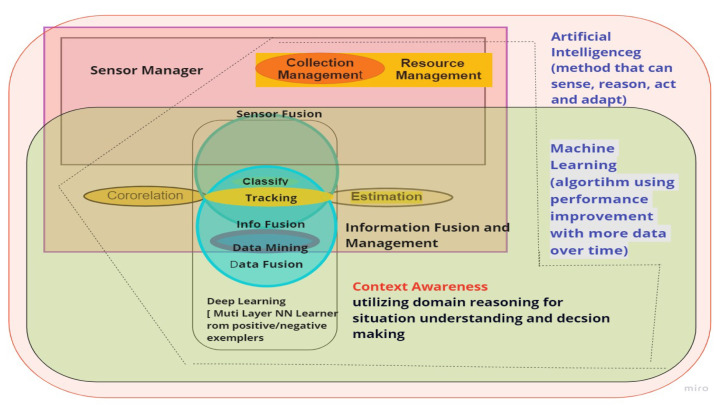
Influence of AI/ML models on data fusion.

Figure 7 illustrates the influence and mechanism behind implementing AI and ML models to fuse multi-sensory vehicular data. Since the 1940s, Chee Chong has emphasized the foundations of control theory, which has sparked an interest in SDF approaches. Due to the lack of computer processing technologies in the 1980s, there were not many companies performing data fusion activities. As the ability to process enormous volumes of data became available in the 2000s, industrial businesses began to investigate big data computing (Figure 4). Many of the current AI approaches have to deal with the issue of companies trying to pack items before conducting studies, evaluating, and analyzing the results, or taking into account the legal and societal ramifications of the discoveries.

The upcoming sections have organized the literature related to data fusion to overcome the multi-modality issue existing in sensory data. Different MSHIF methods indicate various degrees of cogitation from the initial data during the data merging phase. Multi-sensor data fusion uses multiple data fusion techniques at various levels of data abstraction, resulting in the deployment of diverse fusion algorithms. To represent the fusion concepts employed in these investigations, we categorize the fusion methodologies used in various studies. These fusion techniques fall into four groups: Data fusion strategies based on discernible segments, feature complementarity, fusion strategies based on destination attributes of many sensing devices, and fusion strategies based on opinions generated by various sensors. Several articles related to the heterogeneous datafusion of AV sensory data are analyzed on various aspects based on the four categories.

There are some preliminary tasks that have to be achieved for a clear perception creation of the AVs. The basic tasks are

1. Object Detection: The AVs must understand both stationary and mobile objects. The AVs use advanced algorithms to detect objects such as pedestrians, cycles, etc., for effective decision-making. Traffic light detection and traffic sign detection are also a part of object detection. Many algorithms use predefined boundaries (or) boxes to detect objects; 2D object detection uses parameters such as (x, y, h, w, c) whereas 3D methods use more parameters, such as (x, y, z, h, w, l, c, θ).
2. Semantic Segmentation: The main objective of semantic segmentation is to cluster the pixel values and 3D data points obtained from multiple sources into a useful segment that gives appropriate meaning to the context.
3. Other tasks: Other vital tasks include object classification, depth completion, and prediction. Classification determines the category from the point cloud data and image data fed to a model. While depth detection and prediction estimate the distance between every pixel of an image and the 3D point cloud data of a LiDAR sensor.

#### 3.1.1. Image Representation

AVs acquire data from multiple sources such as sensors, cameras, etc, hence a multimodal data characteristic is experienced by the AVs. Since the majority of data collected by the sensors are image representations preferably in 2D or 3D point cloud data formats, adequate procedures must be followed for suitable transformations to process further. Some of the frequently used image representations for the AVs are listed below.

1. Point-based Point Cloud Representation: The data acquired from LiDARS are in the form of 3D point cloud data. The raw data from the majority of LiDARs is often quaternion-like (x, y, z, r), where r denotes the reflectance of each point. Variable textures result in different reflectances, which provide more information in a variety of jobs. Some methods use the 3D points directly obtained from the LiDARs using point-based feature extraction methods. Due to the redundancy of information and slow execution experience by quaternion methods, many researchers transform the 3D point cloud data to voxel (or) 2D projection before feeding them to higher modules that perform complicated tasks related to the creation of perception in the AVs;
2. Voxel-based Point Cloud Representation: In this method, the 3D data obtained from the CNN models are discretized by transforming the 3D space to 3D voxel data. Here $X_{v}=\left\{x_{1},x_{2}\ldots x_{n}\right\}$ where $x_{i}$ stands for a feature vector $x_{i}=\left\{s_{i},v_{i}\right\}$ where $s_{i}$ stands for the centroid of the voxelized cuboid while $v_{i}$ stands for statistical information;
3. 2D mapping based Point Cloud Representation: In this approach the 3D point cloud information (x,y,z) is projected into a 2D space (u,v). Many works use a 2D CNN backbone to perform this transformation. The drawback of this approach is the information loss obtained when 3D to 2D transformation is done.

#### 3.1.2. Fusion Strategy Based on Discernible Units (FSBDU)

This category deals with data level fusion or FSBDU. In this strategy, data collected from indistinguishable sensor units are collected effectively and fused. The fused data is further processed for more accuracy [37]. FSBDU is frequently employed for image enhancement in multi-source image fusion, especially when using remotely sensed image data by fusing infrared and RGB pictures. The longer wavelength makes it difficult to instantly create an image using the data from the MMW-Radar. LiDAR has better spatial resolution than MMW-Radar, although optical images still have a much higher horizontal and vertical resolution. It is also required to adjust them appropriately during regular intervals of period and capacity because the sensor’s FOV and sampling rates differ. Different sensor frames must be aligned because the different information standards and the sizes used by the devices that handle data from various sensors (also referred to as a frame) differ. In the context of FSBDU, space collaboration demonstrates that the same object observed by various sensors correlates to a single reference frame. MMW-Radar imaging has received some attention in recent years in several research [38,39], but it is still insufficient to identify numerous targets in complicated settings. Some investigations use raster maps created by radar or LiDAR that are then combined with optical pictures; this technique is also known as FSBDU methodology. FSBDU is generally separated into two research orientations in the procedure of the sensors (radar (or) LiDAR) hooked with the camera. One creates a raster map using fusion based on location and is based on the findings of obstacle recognition by the radars or LiDARs. A different approach is to use visual imagery techniques as actual samples to create radar or LIDAR images using GAN [40,41].

According to Ref. [42], the AVs environment is represented using sensors such as radar, LiDAR, a camera, and GPS with a map. A grid map is produced using numerous assortments of observed LiDAR data. When the number exceeds a certain threshold, a risk notice will appear for each grid’s empirical threshold of observed values. The identified objects and the candidate objects discovered by MMW-Radar will be compared. The region will be incorporated into the static maps if both of them demonstrate that the target exists. In order to construct a safe driving area and update the vehicle’s location inaccuracy, distance information is employed finally. Reference [43] employs a deep learning technique to locate roads by fusing a LiDAR point cloud with a camera image. The camera image plane is projected over the unorganized dense point cloud to create a series of dense 2D images of the geographical data, which is converted to another format for road separation. In addition, a brand-new conditional multi-generator generative adversarial network (CMGGAN) is presented in Ref. [44], which utilizes data from the radar sensor and the trained model to immediately generate an image of the environment, fully utilizing the entire contextual attributes that the radar sensor has detected. Based on this, FSBDU can be performed as described in Ref. [42] by integrating the produced image with the optical image. A twin static FMCW radar system was suggested in Ref. [45], and it was built by utilizing a straightforward wireless synchronization method and a broad-band omnidirectional antenna. To completely illustrate the superiority of MMW-Radar in penetration, it delivers high-resolution images that are comparable with the images generated by Frequency Modulated Continuous Wave Radar imaging systems to find objects hidden inside walls. Any type of data can be utilized directly to create images with the help of a generative adversarial network (GAN), which can also use the data previously acquired to produce more data of higher quality. According to Ref. [46], it was assumed that the LiDAR or MMW-Radar required a significant amount of computer power to integrate with the camera. As a result, the KITTI data set was utilized to demonstrate the effectiveness of real-time vehicle detection and uses conditional GAN to recreate vibrant contextual image frames from the LiDAR point cloud under the picture’s oversight.

Similar to this, a LiDAR-based character recognition system that replaced the conventional characteristic learning model based on spatial symmetry of the drawing construction process was proposed in Ref. [47]. Additionally, unsupervised location identification is accomplished by using the mapping output of SLAM. According to Ref. [48], directly splicing the slightly higher compared point-cloud data of LiDAR with the picture as the source of AlexNet ensures that the CNN input integrates the image of full information and produces more precise findings. Their system correctly detected all bicyclists and pedestrians at 100%, more than 97% of automobiles, and only 88.6% of trucks.

Similarly, Ref. [49] analyzed RGB and depth data to see whether they can provide relevant information to guide the autonomous vehicles. The authors suggested an innovative data fusion algorithm that meritoriously fuses the raw data for further processing. The authors fused the data using three stages. Raw RGB and depth data are fused in the first stage, CIL architecture input is fused in the second stage, and RGB, D, and CIL architecture output maneuver commands are fused in the third stage. Their inferences suggest that in order to guide the autonomous driving, the multimodality data will often work. Their simulations reveal that decisions made from single modal data provide 46% of accuracy whereas fused multimodal data provides 56% of accuracy in the decision-making of the AVs.

Guan-Horng Liu et al. [50], have proposed a versatile model, namely Sensor Dropout, to improve multisensory policy robustness and handle partial failures in the sensor set. Sensors usually fail when they perceive data other than the data supported by them. To minimize this failure, a policy-switching mechanism is proposed by the authors. In the AD process, multiple targeted hybrid image pixel-level fusion typically makes use of the remediable parts of radar and LiDAR or produced images and then extracts the target parameters and environmental features from the fusion data for further decision-making. Without performing extensive information extraction, FSBDU simply blends the data. Although data collected from several sensors are fused as much as feasible, the data redundancy existing in the fused data further reduces the efficiency of the fused data.

Athma Narayana et al. [51] have suggested a temporal fusion architecture, namely Gated Recurrent Fusion (GFRU) in which they fuse large sensory data and analyze the data further for effective processing. Their model collects sensor data to analyze the drivers’ behavior for effective decision-making. Their proposed architecture produces an optimal fusion strategy for every time interval to select the best data fusion approach. GFRU does away with the requirement to create and train distinct network blocks for acquiring transitional abstractions, cleaning sensor input, and understanding driver behavior. The study attempts to see how well GRFU works in other difficult temporal multimodal contexts that are not related to autonomous driving.

Yi Xiao et al. [49] have analyzed RGB and depth data to see whether they can provide relevant information to guide autonomous vehicles. The authors have suggested an innovative data fusion algorithm that meritoriously fuses the raw data for further processing. The authors have fused the data using three stages. Raw RGB and depth data are fused in the first stage, CIL architecture input is fused in the second stage, and RGB, D, and CIL architecture output maneuver commands are fused in the third stage.

#### 3.1.3. Fusion Strategy Based on Complimentary Features (FSBCF)

With the use of fused multiple sensory features, the FSBCF technique, based on the desired features, accomplishes classification and recognizes multi-target characteristics that have been retrieved from matching sensor data. Since uncorrelated dimensions of the same target can be captured by diverse sensors, it results in better identification of target detection and recognition. The intended characteristics and data features are two of the retrieved features in the AD system.

1. Target parameter extraction: From the pre-processed data, it extracts target information such as dimension, range, orientation, velocity, and acceleration. Many studies create regions of interest (ROIs), which immediately translate the position of the object detected by the radar into an image to produce a region, extract location characteristics from radar or LiDAR targets, and aid image identification;
2. Data feature extraction: The main objective of feature extraction is to separate the needed features from all formats of processed data to classify and detect the objects. A few examples are color, shape, edge, spectral frequency, texture, velocity, latitude, longitude, etc.

Therefore, in order to produce fewer ROIs, many studies first extract the target’s distance and azimuth information using radar and LiDAR and then integrate that data with the image data. In order to precisely classify the target’s category and further pinpoint these areas of interest, the pre-trained model is applied. Many studies used machine learning techniques for additional perception tasks after obtaining ROIs. In order to categorize these features using SVM, AdaBoost [52], and other techniques, traditional machine learning approaches typically first extract common features from pictures using the Haar operator, HOG operator, and gray-level co-occurrence matrix (GLCM). In recent studies, neural networks such as YOLO, CNN, and ANN are frequently used to classify and recognize targets. According to Ref. [53], the use of a near-infrared camera and radar allows for the accurate, dynamic identification of humans on platforms for AD vehicles. The cascade-improved classifier makes it simple to combine the radar and camera data with the feature layer. The time-frequency spectrogram of human activities is employed in radar-based human motion recognition, and the short-time Fourier transform (STFT) is a common technique for examining time-frequency properties [54]. Due to the differences in the objects’ dimensions, the features collected for identical destination objects from several sensors increase the detection performance of the target object. However, the deep learning-based approach is more advantageous in terms of recognition accuracy and does not require a process for artificially extracting features. Without the artificial feature extraction procedure, the neural network architecture must have a fusion process based on complementary characteristics. As a result, with the advent of deep learning, the analysis of an attribute-oriented fusion of heterogeneous sensory data has all but ceased. Recently, FSBCF has relied heavily on the positional properties of MMW radar and LiDAR for complementary fusion.

Another innovative technique to obtain sequential characteristics for atomizing movement categorization is the long-term and short-term memory (LSTM) unit superposition recurrent neural network (RNN) in Ref. [55]. A method of MMW-Radar and camera fusion sensing was suggested in Ref. [56]. The corresponding picture regions are created from the radar coordinates, which also include distance and angle information, and the ROIs are then categorized using the deformable part model (DPM). The detection accuracy for the recognition result was 98.4%. Additionally, LiDAR was used to create the potential target coordinates in Ref. [55]. After creating ROIs from point cloud pictures and performing 3D target identification and categorization, Ref. [57] implemented a high-level region architecture to describe targets in 3D form. To segment the road in front of the car, Ref. [58] proposed a composite stochastic domain architecture based on the conditional random field model, integrating camera, and LiDAR features. The KITTI-ROAD benchmark dataset has been the subject of a significant number of trials, and the results demonstrate that this approach outperforms the present approach. It is important to note that Ref. [43] uses a complete CNN model to process the LiDAR and camera inputs (FCN). Early fusion strategy (EFS), late fusion strategy (LFS), and cross fusion strategy (CFS) were utilized to merge LiDAR and camera data, and FCN was used to create safe driving zones on existing roadways. There are 21 layers in the FCN as a whole. LFS inputs the first 20 layers with 2 distinct kinds of information and merges 2 distinct output values in the final layer. A weighting concept is introduced by the CFS with successive data layers and is fully fused at the last layer. LiDAR and camera signals are intrinsically linked by EFS to create a six-dimensional tensor, and the information is then trained using FCN to provide a safety zone. By implementing various fusing techniques, it is possible to fuse different formats of data successfully. However, we cannot conclude that the separated data can have distinct networking data obtained from the fusion techniques, due to the huge volume of data collected from the sensors. A significant amount of information related to the extracted features from the raw data is necessary for FSBCF in order to blend the statistically independent features or parameters found in various sensors. Higher dimension features are better at distinguishing between targets, increasing the effectiveness of the fusion, and overcoming the limitations of a single feature. Due to the direct application of the existing neural network design for visual pattern recognition, there has not been a significant study in recent years on merging the features of multi-sensor systems. The majority of research focuses on the target parameter extraction technique to implement the FSBCF fusion strategy.

Ref. [59], have proposed an innovative fusion model that covers both early and late fusion. The authors select the best classification model using advanced ensembling and gating techniques to improve the accuracy of the fusion. The authors also propose and evaluate both static and deep learning-based context identification strategies. In another similar study, Ref. [60] collected data from the forward-facing camera and LiDAR sensors fixed in AVs and effectively fuse them to improve the contextual accuracy of the AVs. The author’s approach helps AVs identify objects during the winter season when the visibility of the roads is not clear due to snow and fog. The authors have used seven models to perform all types of fusion (early, intermediate, and late) with all combinations of the perceiving devices.

#### 3.1.4. Fusion Strategy Based on Target Attributes (FSBTA)

A distributed fusion methodology based on target attributes (FSBTA) uses every sensor to gather target attributes and identify various targets to create a target list. To obtain accurate, trustworthy targeted information, and prevent anomalies and missed inspections, multiple target lists will be combined. Multiple cameras, MMW-Radar groups, and LiDAR are used in Ref. [61] to obtain information from the vehicular environment and produce the matching destination lists. The destination lists that match allow for the planning of a safe driving area for AD vehicles to reduce the possibility of collision. ROIs are created for the image in Ref. [48] using the MMW-retrieved Radar’s overall performance, and a CNN model is then used to locate the destination inside the ROIs. In the meantime, the objective lists viewed by the camera and MMW-Radar, respectively, were compared and integrated. Destination type, proximity, velocity, position, and angular velocity are all included in the merged information. The fusion result increases resilience and to some extent withstands the missing recognition of a single sensor. According to Ref. [62], the motion characteristics of several targets are extracted using the 2D Fast Fourier transform (2FFT) and scattered attribute identification in a radar and visionary component, respectively. In addition, the divided entities are tracked using the Gaussian inverse Wishart probability hypothesis density filter (GIW-PHD). According to Ref. [63], goal objectives produced by LiDAR, MMW-Radar, and camera are finally combined. Low-level information fusion was carried out by LiDAR and a camera, and they subsequently utilized the range and angular information of LiDAR to build ROIs in alternate visualization. The redundancy between the sensors is managed through target fusion. The camera provides high levels of 2D data such as hue, luminosity, concentration, and texture features while LiDAR provides 3D point cloud data. Compiling as many attributes as is practical, enables the interpersonal relationship between humans and computers followed by intent identification. For the purpose of detecting pedestrians, Ref. [64] applies the fusion processing of LiDAR and camera sensors. Additionally, the 3D point cloud data is used in this study to determine the target’s shape in order to lower the pseudo alarm rate and address the object obstruction issue when detecting the individuals using cameras. In this study, ROIs for the image is created using LiDAR target information. In order to accelerate pedestrian detection and achieve mean accuracy results of 99.16%, the objectives identified by LiDAR data and photos are compared simultaneously. Ref. [65] detected the front vehicle’s lane change behavior using a stereo camera and LiDAR. They used a NN model based on particle swarm optimization to categorize the proximity, tangential velocity, and longitudinal velocity of the vehicle to determine the line of traffic attitude, and the overall classification score was over 88%. The degree to which the data are abstract after integration by FSBTA is somewhere between FSBCF and FSBMD. This type of fusion technique employs several sensory devices to detect the objects and accordingly combines the object qualities or ambient variables that are extracted. This fusion technique will enhance the perception system’s stability and dependability in order to deal with the possibility of false alarms or missed detections when individual detection and recognition are conducted on a single sensor. Ref. [66] have proposed a study to detect 3D objects in the autonomous driving environment. The authors propose a multiview 3D network (MV3D) and a sensory fusion architecture that uses LIDAR point cloud data and RGB images of the surrounding environment as inputs to generate 3D boundary boxes. To encode the 3D point cloud data, the authors use an advanced encoding scheme. Furthermore, they developed a deep fusion methodology that allows interaction between intermediate layers of different paths by combining region-wise features extracted from multiple views. The author’s proposed method outperforms existing LiDAR-based and image-based solutions. However, the optimization time of the proposed model is high, which thus reduces the efficiency of the fusing model.

In yet another interesting study, Namen Patel et al. [67] proposed a novel end-to-end system for autonomous vehicle navigation, based on decisions made by combining raw pixels from a front-facing camera with depth measurements from LiDAR. Even if the sensor fails, their proposed networking model effectively conducts modality fusion and predicts steering commands. The proposed architecture consists of three networking layers: NetGated, NetEmb, and NetConEmb, which make up the CNN model’s convolutional layers.

Ref. [68] proposed a fuzzy logic-based data fusion technique. The model proposed by the authors uses magnetic sensors to estimate the occupancy probability of the relevant parking space. The model removes, the bias of the previous model, but the scope of the study is minimized due to complicated fuzzy rules. Ref. [69] investigated the functionality of a multimodal vision sensor that combines data from three types of cameras: stereo, polarization, and panoramic. Each sensor provides information on a particular dimension. The authors have proposed a CNN model, namely ERF-PSPNet, to effectively segment the image data by using advanced encoding and decoding techniques. Their suggested multimodal sensor has already been applied in several intelligent vehicle systems for nighttime semantic comprehension, visual topological positioning, and panoramic image parsing.

Another interesting trust evaluation model proposed by Ref. [70] fuses the GPS data and the contextual information of the multiple AVs to ensure that the vehicles are close to each other. The authors use distance metrics and clustering techniques to accomplish their study. The authors in Ref. [71] proposed a Global Navigation Satellite System (GNSS) spoofing attack detection framework for AVs. The authors collected data from minimal-cost internal AV sensors (accelerometer sensor, steering angle sensor, speed control sensor, and GNSS), and fed them into an LSTM-based recurrent neural network to forecast the positional change, which is the distance traveled by the AV between two consecutive time stamps. The information obtained is compared with the GNSS-based location shift, to detect the attacks.

#### 3.1.5. Fusion Strategy Based on Multi-Source Decision (FSBMD)

FSBMD determines the target’s location, characteristics, and categories using only one sensor’s data. It then uses a particular fusion technique to accumulate the choices made by several sensors, and the necessary techniques are used to arrive at the final fusion result. The fusion effects have a significant effect on determining whether the final choice outcomes are correct., and FSBMD integration directly creates decisions for specific aims. Rule-based fusion, decision-making, fusion based on trustworthiness, and probability fusion are typical subcategories of FSBMD [72]. Subjective Bayesian probabilistic reasoning, Dempster-Shafer (D-S) evidence-theory-based reasoning, artificial intelligence (AI), and fuzzy subset hypothesis are among the approaches used by FSBMD. In Ref. [61], a framework with numerous sensors was established for the AVs. This platform is used to extract, through data processing, the target’s motion characteristics, road limits, lane markers, traffic signs, and barriers. Controlling the mobile status of AD vehicles can be accomplished by using a rule-based strategy based on the system’s training data. For a heterogeneous data-based vehicle classification system, an ANN post-fusion technique was suggested in Ref. [73]. This system used optical image, 3D LiDAR range data, and spectral response data as three distinct methodologies, each with its own identification process. Joint re-scoring and non-maximum inhibition were used to combine the results of each modality. Additionally, FSBMD’s object recognition effectiveness has increased by 1.2% points in comparison to the individual modal. A framework for fusing thorough fuzzy theory with the nervous system was put forward in Ref. [63]. The author’s proposed architecture helps create an effective knowledge combination system to identify the navigation framework, by combining Kalman separation and fine processing requirements. New concepts for developing data collection models, workflows, selection, and analyzing methods are offered by fuzzy sets. Among the most significant foundations of the neural system is the adaptive neuro-fuzzy inference system (ANFIS). ANFIS is an effective instrument to control experimental inconsistency in any model and also has high acceptance and predictive power [74,75]. In addition, a framework for fusing evidence based on the D-S evidence theory is suggested in Ref. [76] to deal with the targets’ uncertain mobility and the noise-proneness of sensor data. The target is then classified, particularly for pedestrian detection, using a classification index that is established by combining a reliability function with the measured value. Additionally, when the uncertainty model is erroneous, the assured characteristics in the study change the probabilistic approach provided by the sensor to enable trustworthy decision-making. Ref. [77] suggested a localization algorithm by combining a camera, GPS, and onboard sensors for precise vehicle placement. They combined GPS data with ocular odometry using the extended Kalman filtering algorithm, increasing the accuracy above conventional GPS positioning techniques by 40%.

The final fusion impact is determined by the performance of the fusion strategy, in which FSBMD synthesizes numerous judgments made by several sensors. This level of data fusion resulted in the direct production of Refs. [78,79], which was the ultimate conclusion. By using this approach, it is possible to successfully prevent the ambiguity and unreliability that can occur from making a final judgment based solely on the perceptual results of one sensor. Data complementarity is also quite low, and FSBMD does not considerably enhance the efficiency of object identification at the computational level. This tactic was frequently used with others in various research. Table 3 lists the particular tasks completed by a variety of sensors in diverse investigations. Similarly, Table 4 depicts the datasets that are frequently used by researchers to evaluate their contributions related to AD.

Table 5 illustrates the chronological summary of various fusion strategies along with their pros and cons. The tick mark indicates that the drawbacks identified in the previous study are rectified by the current study organized in chronological order.

### 3.2. Situation Awareness

Fusing the raw multimodal sensory data improves the accuracy of the environment perceived by the AVs.This section discusses the basics of contextual awareness in AVs and the influence of data fusion on the accuracy of conceptual awareness. There are several models used to measure the situation awareness of AVs. The two major challenges associated with launching AVs in real-time environments are (1) the need for robust, self-diagnosing, and explainable embedded perception and (2) the need for understandable driving decisions. Most of the SA models use the following approaches to perceive environmental information. The first approach is Embedded Bayesian Perception. The main aspects that are covered by this approach are reasoning about uncertainty and time window using past and future events, improving the robustness using Bayesian Sensors Fusion, interpreting the dynamic scene using Contextual and Sensor Information, and software and hardware integration using GPU, multicore and microcontrollers. Multisensory data is collected using LiDAR, radar, and stereo cameras. It is a probabilistic environment model including dynamics. The model provides a clear distinction between static and dynamic environments. The model is designed for highly parallel processing. It includes embedded models for motion prediction and collision risk assessment. The second approach is a dynamic probabilistic grid and Bayesian Filtering, which is exclusively used to exploit dynamic information for a better understanding of the scene. Mica. R. Endsley have analyzed various factors related to AVs, where SA plays an important role. They found that although it is difficult to collect clear vehicular data, some useful information can be grabbed from the disengagement reports collected from the manufacturers testing the vehicles. Some of the disengagements include (i) manual takeovers by the human drivers completely disengaging the automation facility, and (ii) charges taken by the humans when the software that helps in automation completely disengages itself.

From Table 6, it is evident that Waymo changed from automated mode to manual mode after covering approximately 5000 km [136]. From the results, it is apparent that further analysis has to be done by the leading manufacturers before launching level 5 autonomous vehicles. Common disengagements occur due to the following reasons.

**Figure 8:**
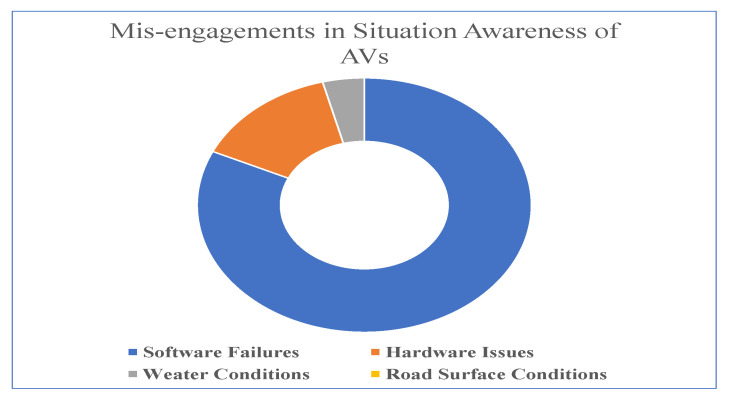
Percentage of failures due to misengagements in AVs.

The above failures are related to a specific region to a particular place where the testing is performed. However, the scenarios might change if the test is carried out in yet another place (or) under different environmental conditions. Most of the failures in the AVs happen due to a lack of collection of proper environmental information. Situation awareness is significantly reduced by automating several tasks in the AVs. Some of the major reasons for SA failures are increased trust in human monitors over automated vehicles, limited information perceived by the AVs as a result of deliberate or accidental design choices, and reduced level of information coming from passive processors rather than active processor information. Passive processing of environmental data can still lead to poor SA, transforming drivers to act as passengers with a lesser understanding of SA. Automation increases SA, but the continuous automation process decreases SA due to the overconfidence of the driver’s over-automation. “The more automation is introduced to a system and the more dependable and robust that automation is, the less likely that human operators monitoring the automation will be aware of vital information and able to take over manual control when needed”, as stated by the automation paradox. This conclusion is based on a number of studies that suggest that as mechanization becomes more proficient, the public is paradoxically more inclined to lose SA and trust it. Furthermore, when more functions are automated and the hierarchy of mechanization develops, their SA is harmed, making them relatively ineffective at manual monitoring and intervention. SA in AVs is established using advanced AI techniques that develop with time and helps to observe analytical connection in data and also find the relationship between the identified features of the surroundings with set performance outcomes. According to AI expert Perl, developing these enhanced systems is very difficult and several trial-and-error techniques have to be implemented with their techniques to obtain the actual result. Figure 8 depicts the failure rate due to irregular engagements caused in the AVs due to its poor perception. Similarly, Ref. [137] explored various methods to measure the SA in AVs. Some of the techniques used are Eye Tracking, which is level 1 tracking, measured by the drivers as what they observe from the road, and Physiological measures, which use psycho-physiological measures such as measurements of brain activity and blood flow and relating these observed measures with the environment data. The Situation Awareness Global Assessment Technique (SAGAT) involves halting the current simulation and further querying the person to acquire various information such as position, type, and future status of the elements within the scene. Another method is SART (Subjective Ranking Technique), in which the drivers rank themselves or by the observers, their behavior, and decision in driving according to their perceived environment information. Similarly, another method, Question Probes, provides objective measures of elements perceived in the environment and also addresses levels 2 and 3 of situation awareness. On account of the above discussion, Ref. [138] proposed an innovative Eye Tracking method to aid in understanding how visual engagements in non-driving related tasks affect changes in SA of the driving environment after a takeover request. Ref. [16] discusses various factors related to SA in remote operation and also highlights the advantages and disadvantages related to SA in both manual and automated driving.

Ref. [139] have proposed AI/ML models to improve the SA of the AVs by comparing the image frame with the previous frames. By correctly adjusting the analytical neural networks and applying late fusion methods, they suggest unique multi-modal systems that achieve robustness to adversarial attacks. To be more precise, the authors suggest a comprehensive strategy that strengthens the robustness of a 2D segmentation DL model to adversarial noise by adding new layers to it. Then, using a cutting-edge late fusion technique, direct features were extracted from the 3D space and projected into the 2D segmented space to look for discrepancies. The KITTI odometry dataset has been extensively evaluated, and the performance results are encouraging. Fusing the data by extracting the salient features has improved the accuracy of their models. The CNN models produced 72% accuracy and the PointRCNN model produced 78% accuracy. Ref. [140] proposed an innovative strategy to measure the SA of the humans inside the AVs. Their model created a semantic pattern of human awareness and vehicle state to estimate the SA, based on which effective decisions are taken by the AVs to maintain a safe trip. This survey has analyzed all these facts and further explored how multimodal data fusion improves the SA of the AVs, which further has an impact on the accuracy of decision-making in the AVs. Eminent articles from different reputed sources are analyzed in detail. Articles representing the key areas are further grouped based on different pointers (a purpose to derive a strategy to address a specific problem) and methods.

The architecture consists of three stages, namely: Firstly, Unimodal architecture, which gives results for each information signal’s baseline segmentation separately. Secondly, VGG (convolutional neural network model), which is used in this encoder-decoder architecture. To reconstruct the original picture, the VGG’s normal layers are replaced with three up-sampling layers. Thirdly, Early-Fusion architecture, which uses CNN to remove joint features by fusing two signals prior to potential extraction and Multimodal Mid-Fusion Architecture, which fuses color, motion, and depth information altogether using the Mid-Fusion approach. For increased performance, future studies could entail proposing a more complicated network architecture that supports all three information cues. Further structural restrictions can be integrated to improve segmentation outcomes.

Similarly, in yet another interesting study, The importance of motion, depth, and color for taking important decisions in autonomous vehicles are explored by Ref. [134]. Depth cues are used to detect the road conditions, and motion cues are used to analyze the dynamic environmental scenarios such as the movement of objects such as vehicles, pedestrians, etc. The authors’ proposed research improves picture quality by using geometric details modeled by depth maps and motion cues, which simplifies the decision-making even further. To get the most out of all details, the authors propose a CNN architecture that fuses depth, color, and motion using their proposed fusion algorithm. The CNN architecture extracts features from three input signals before performing feature-based fusion. The encoded output is up-sampled to reach the final image scale, based on which autonomous vehicle navigation decisions are made. Early and Mid fusion have improved the accuracy of the AVs context to a great extent. Greater improvement was attained for several classes, such as the Truck, Van, Building, and Traffic Lights classes, which had improvements of 32%, 28%, 9%, and 8%, respectively. The authors improved the mean IoU with imperfect depth maps by 3% compared to RGB-only. Further, they utilized flowNet as a realistic flow estimate for optical flow and ground truth as the best estimator for other types of flow. Moving classes were greatly enhanced, while mean IoU improved by 4% as a result of fusion with ground truth. For the Truck, Van, and Car classes, improvements of 38%, 28%, and 6%, respectively, were attained. Fusion with FlowNet raised the average IoU by 2.37%. Early fusion is denoted by RGBD, mid-fusion is denoted by RGB + D, and ground truth is denoted by GT. A similar approach is used for optical flow fusion.

### 3.3. Decision-Making

The final decision-making in AVs is one of the important tasks. Decisions are made on various events related to AVs. For reliable and accurate instant decision-making in AVs, clarity in the fused data is essential. Hence, much importance is given to data pre-processing and data fusion tasks. Autonomous vehicles are anticipated to alleviate road congestion, reduce collisions and fatalities, improve flexibility for kids, senior citizens, and the disabled, and eliminate the demand for parking space in cities. An autonomous vehicle’s planning strategy fulfills three tasks: mission planning, in which the vehicle resolves a navigational issue to accomplish a task, decision-making, in which the automobile selects an appropriate action for the next time step from a set of options, and path planning, in which the vehicle predicts its future course as a consequence of time or space. The survey has grouped decision-making strategies proposed by various eminent research scholars based on various events such as lane changing, rule-based decision-making strategies, collision avoidance, platoon management, pedestrian crossing, congestion avoidance, highway overtaking, and decision-making in uncertain conditions.

Decision-making articles related to both manual and autonomous vehicles are reviewed to explore various innovative decision-making strategies suggested by various researchers. Contents are gathered and reviewed based on decision types, methods, pointers, and events. Decision types fall under three categories, namely sequential, actions, and end-to-end. The sequential approach uses advanced visualization techniques in decision-making processes to plan and control autonomous vehicles. Decision-making and preparation are incorporated in interactive planning to control the behavior of the vehicles, and items of interest are detected and fused into a scene summary using an end-to-end strategy, based on which driving commands are computed. Pointers represent various types of research articles that highlight the importance of the decision-making process, while methods correspond to the various empirical, statistical, and machine-learning approaches used in decision-making strategy. Finally, the fourth category of taxonomy summarizes various research articles that have proposed innovative strategies in decision-making to overcome various roadside events that are faced by autonomous vehicles. The below paragraphs summarize the major contributions and drawbacks in the existing literature related to decision-making in autonomous vehicles.

#### 3.3.1. Lane Changing

An interactive behavior-generating and responding game theoretic architecture has been suggested by David Isele et al. [141]. Their primary contribution is to demonstrate how an AV can use a game-tree decision-making approach, including approximations and justifications to computationally simplify the tree search. Teng Liu et al. [142] have designed a controller that focuses on the safety and performance of AVs. The modeling of the overtaking plot is addressed first, and the comparative strategies known as the smart driver model and the minimization of braking scenarios caused by lane changes are defined in the later stages. For highway overtaking decision-making, the Dyna-H algorithm is used, which blends the modified Q-learning algorithm with a heuristic planning approach. The outcomes demonstrate that the suggested decision-making approach could give superior results in convergence rate and control. Yonggang Liu et al. [143] have developed an innovative lane-changing model for AVs. The authors used various parameters such as benefit, tolerance, and safety to decide whether autonomous vehicles can change their lanes or not. Since nonlinearity exists in the model due to multiple parameters, the study uses SVM and Bayesian optimization to solve the problem. For connected autonomous vehicles, Jianqiang Nie et al. [144] proposed a decentralized cooperative lane-shifting decision-making system (DCLDF). The authors have suggested a system for autonomous vehicles to make successful lane-changing decisions. The system is made up of three main modules: state prediction, candidate coordinates, and candidate decision. To predict the current state of related cars, the state prediction module uses a cooperative car-following model.

#### 3.3.2. Collision Avoidance

Constantin Hubmann et al. [145] proposed a partially observable Markov decision process for AD. It generates near-optimal behavior on intersections with unpredictable layouts and a diverse count of traffic respondents who have uncertain maneuver intentions. This research looks at two highly fascinating features of other road users’ uncertain predictions. This work’s central aspect is the representation of an online Partially Observable Markov Decision Process (POMDP) for AD. Jimim Rhim et al. [146] suggested a system to examine human moral reasoning ability in order to frame guidelines for autonomous vehicles to make successful collision avoidance decisions. They looked at driver actions in two countries: Korea, which has a strong communal civilization, and Canada, which has a typical independent civilization. Real-world crash scenarios are produced first, followed by interviews with both courtiers. Three types of human conduct clusters are generated based on the data collected: Moral Altruist, Moral Non-deterministic, and Moral Deontologist.

Ref. [94] proposed a modern Dedicated-Short-Range-Communication (DSRC) inter-vehicle communication system along with vehicle localization-based rear-end collision avoidance methods, to help the AVs avoid rear-end collisions. Hongliang Yuan et al. [147] suggested a decision-making strategy for autonomous driving to avoid traffic collisions. The authors used a unique optimization technique to effectively decide whether autonomous vehicles should apply straight-line brakes or steer to change direction during inevitable crashes. They tackled collision avoidance directly from the standpoint of limited progressive optimization.

Table 7 illustrates the chronological ordering of articles related to lane changing and collision avoidance of AVs. The tick mark indicates that the drawbacks identified in the previous study are rectified by the current study organized in chronological order.

#### 3.3.3. Multiple Decisions on Roadside Events

Dimia Iberraken et al. [148], using a Sequential Level Bayesian Decision Network (SLBDN) and an effective empirical formalization of parameters for anemology detection based on a Dynamic Predicted Inter Distance Profile (DPIDP) between vehicles, suggested a probabilistic overall strategy for risk evaluation and management of AV in highways. The system proposed by the authors takes an appropriate decision on the current risk scenarios and takes appropriate actions to help autonomous vehicles to find alternate solutions to overcome the obstacles. The crash data obtained between 2016 and 2017 from GIS open data held by the Nevada Department of Transportation, city of Las Vegas, was analyzed by DaiQuan Xiao et al. [149]. The results of the analysis by the authors summarize that buses are the major sources of accidents, as they have contributed to 466 crash occurrences. Alkis Papadoulis et al. [150] developed the Connected Autonomous Vehicles (CAV) algorithm and implemented it using the VISSIM simulation tool. Their proposed application interacted with an interface, namely the External Driver Model Application Program Interface. Real-time data is used by the simulated model to effectively frame the rules and associated actions, which are applied when the vehicles cross the lanes or brake due to instant conditions. Christopher G. Burns et al. [151] used the concepts of Human Machine Interface, and analyzed the behavior of autonomous vehicles when humans cross their normal routes. The authors have tried and experimented with various possibilities of pedestrian behavior and accordingly framed rules based on which decisions are made by autonomous vehicles. Their approach needs more research into the ideal communication methods between AVs and pedestrians. Changxi You et al. [152] suggested a method to assist autonomous vehicles in making appropriate decisions to respond to multiple roadside events. The proposed model of the authors used a Markovian decision process (MDP) to create a relationship between autonomous vehicles and their environment, with the expert driver’s driving style as the goal to be mastered. The MDP uses road geometry to learn various driving styles from the drivers. To measure the MDP’s reward function and estimate the drivers’ driving behavior, reinforcement learning strategies are used.

Jaime. F. Fisac et al. [153] have suggested a system using a game theory approach for autonomous vehicles to predict the vehicles and driver’s behavior for taking effective decisions to overcome various events such as lane crossing, accidents, and congestion. To handle the mutual impact between a human and an AV while keeping computational tractability, the authors have developed a multilayered game theory paradigm. Their framework has suggested a coupled interaction model, which accurately predicts the change in the driver’s behavior during multiple seconds. Table 8 illustrates the chronological ordering of the referred articles related to decision-making for multiple roadside events. The tick mark indicates that the drawbacks identified in the previous study are rectified by the current study organized in chronological order.

Similarly, appropriate decision-making in the formation of platoons in AVs is another challenging task. Various innovative models and ideas have been proposed by eminent researchers for efficient platoon management in AVs. Refs. [154,155,156,157,158,159,160,161] are a few of the current studies in which the researchers have proposed various innovative ideas related to all salient aspects of platoons in AVs. Studies done by eminent scholars, see Refs. [162,163,164,165,166], portray various decision-making strategies in autonomous vehicles. Different ideas proposed by several authors give a new dimension to how effective intelligent decisions are taken by AVs to overcome roadside events.

Table 9 illustrates the chronological ordering of contents related to decision-making for platooning events of AVs. The tick mark indicates that the drawbacks identified in the previous study are rectified by the current study organized in chronological order.

#### 3.3.4. Surveys Related to Decision-Making

Wilko Schwarting et al. [167], have conducted an exclusive survey on various emerging developments and challenges in the area of self-driving vehicles. In their survey, they examine recent developments in the fields of perception, planning, and decision-making in great detail. For decision-making, the studies analyzed in their survey have proposed various innovative strategies. One of their preferred studies [168] uses a partial observable decision-making process (POMDP) to make effective decisions in autonomous vehicles. In POMDP, other vehicles’ intentions are represented as hidden variables. They record the states of various recorded vehicle motions and accordingly plan the paths of the vehicles. Another research [169] used the POMDP technique to combine the highway background and the mobility purpose of other vehicles for effective decision-making in lane changing of the new autonomous vehicle in their survey. By examining the departure from the reference behavior, the responses of the other cars can be deduced, which is specified to conform to the road background. Another intriguing study [170] mentioned in their analysis proposes a unique POMDP solver that proliferates numerous band-defined principles, including hyper-parameters, and calculates the predominant closed-loop repercussions on the AV’s principles for decision-making. The main assumption is to evaluate each sample using a single set of policy assignments while condensing the decision to a minimal number of policies. To compute secure trajectories, the authors used a coupled POMDP to predict the potential trajectory of the interacting traffic participants and a chance-constrained nonlinear MPC planner. In the case of occlusions and imperfect vision, another important article referred to in the author’s survey [171] suggested a continuous POMDP with an emphasis on balancing discovery and exploitation. During value iteration, the continuous POMDP is solved by incrementally learning an effective space representation. To detect potentially unknown objects, the authors took into account the experiences of road users. Although the authors propose innovative strategies for instant decision-making in autonomous vehicles for lane changing, they fail to address new emerging paradigms such as interactive planning and end-to-end learning related to the safety and reliability of autonomous driving.

Table 10 pinpoints the background behind various methods used by the authors to design their suggested decision-making models to frame effective decision rules in autonomous driving.

## 4. Pitfalls and Future Research Direction

This section summarizes the limitations of relevant research literature to the three major areas namely: multi-modal fusion, situation awareness, and decision-making literature related to autonomous driving.

### 4.1. Analysis of Fusion Strategies and Perceived Results

It is challenging to compare which strategy is superior because different studies employ varying statistics for a variety of purposes or situations, and the precise methods employed during the implementation process vary as well. However, FSBDU and FSBCF can best utilize the complementary use of various sensor data from the viewpoint of information fusion. Additionally, several research combines different methodologies in order to further increase the reliability of fusion. Table 3 lists the exact tasks that several sensors in various experiments were able to complete. The ongoing study focuses on two categories for specialized sensing tasks: the detection of dynamic targets and the perception of the surroundings. Moving target perception includes barriers such as bicycles, cars, and individuals, among others. Many researchers only achieve the detection and recognition of the target, while others go beyond and examine the movement pattern of the target, considering the outcomes of the detection. Target tracking loss can be avoided by examining the motion trend since it is challenging to guarantee the reliability of each frame’s detection. The methods for determining an object’s existence and producing its trajectory are covered in many recent articles. Consequently, to control the front driving area of the AV safely, some works merge lane detection strategies with obstacle detection methodologies. Meanwhile, other researchers have based their data visualization on the above-mentioned approach. Visualizing the data is obviously vital to confirm that the safety zones are accurately marked, but it is not necessary for AD at the L4 or L5 levels. When using different sensors for target identification and tracking, a target corresponds to multiple distinct positions in various data because of the varying coordinates that each sensor is positioned in, the data sampling rate, and the range of view (FOV). To receive the target location data after fusion, these coordinate systems must be unified. MMW-Radar and camera calibration as well as LiDAR and camera calibration are the two main types of sensor calibration currently in use. Currently, the camera, LiDAR, and MMW-Radar are generally used in the AD process to accomplish the detection and identification of objects. The addition of additional sensors (such as communication tools, GPS, and IMU) will increase the vehicle’s range of real-time sensing capabilities. To fuse larger datasets, the SVM model performs better than the Bayesian approach.

### 4.2. Decision-Making Models

The accuracy level reached by most of the referred decision-making frameworks and models is not satisfactory for various critical maneuvers and use cases [172]. Moreover, the literature studies have either used fewer datasets with minimum features (or) collected data from minimum sources to evaluate their decision models. Given the limitations of the datasets, it is difficult to gauge the effectiveness of the decision-making approaches described in the literature [143,144,146,173]. Furthermore, most of the relevant research literature did not focus on the ensemble concept and thus is unable to accommodate the newly arriving features or instant environmental changes. Moreover, they suffer from a lack of studying the efficiency and success and failure rates of their proposed decision-making models.

### 4.3. Influence of Data Fusion towards Situation Awareness

**Figure 9:**
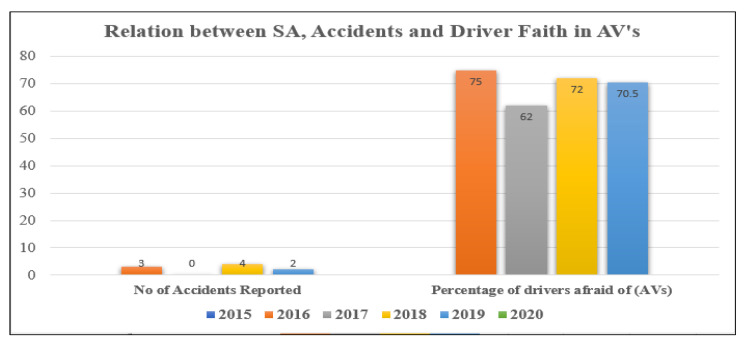
The relation between SA, accidents, and driver faith in AVs [177].

From the overall analysis of articles related to the impact of data fusion on SA, it is identified that most of the works not only focus on the estimation of various factors related to SA but also on multiple ways to represent the surrounding environment of the AVs. Many authors have used advanced mathematical models that involve polynomials and coefficients as states to measure the lane geometry [142,144,174]. The authors have fused the motion information of the ego vehicle and its corresponding neighboring vehicles using cameras and computer vision to improve the estimation of the lane geometry. Similarly, for identifying the road borders and other objects in the AVs environment, most of the studies use mathematical models that use discretization and probability concepts such as grid mapping techniques and quadratic probing concepts, to improve the accuracy of SA [143,175,176]. More pre-processing of the camera and sensor data is required in the referred studies to improve the accuracy of the data, and further efficient utilization of computer vision concepts to study non-planar road conditions will improve the accuracy of the SA. The contribution of various authors towards improving the SA in AVs can consider the difference in height between the camera and the sensor framework, to improve further estimations, especially when there are few radar measurements as input to the system. Parameter and model uncertainty has to be considered to improve the accuracy of the SA. Further SA has an impact on the accidents caused by the AVs, which in turn indirectly declines the faith among the users to use the AVs. Figure 9 clearly illustrates the relationship between the accident ratio and the faith of AV users. Thus, accurate and clear environment data perceived by the AVs help them make proper decisions, resulting in minimizing accidents [177].

## 5. Conclusions

Instant decision-making based on enhanced contextual awareness is crucial to ensure safety in autonomous driving. The clarity in multi-modal sensor data followed by fusing the multi-modal data into a suitable format is an important task for creating accurate situation awareness and decision-making in autonomous vehicles. This survey identified the key concepts related to data preprocessing, preferably multi-modal fusion, situation awareness, and decision-making in autonomous vehicles. Our extensive research literature survey advocates that among others, data preprocessing stands out as one of the major categories for research contributions, owing to the huge potential for enhancements of state-of-the-art solution approaches. In the case of data fusion, many researchers stop at detecting and recognizing the target, whilst others go further and look at the target’s movement pattern. Since it is difficult to ensure the accuracy of frame detection, target tracking loss can be prevented by analyzing the motion trend. Many recent studies tackle the strategies for determining an object’s existence and producing its trajectory. Most of the studies fail to propose a generic multimodal fusion methodology to handle the diversity existing among different datasets. The relevant research literature also did not clearly explain key operations such as feature selection and dimensionality reduction of multimodal data, the mechanisms for 2D to 3D multimodal data transformation and storage, and the methodology for converting multimodal data to a single unique data format. Regarding decision-making, the accuracy level reached by most of the referred decision-making frameworks and models is not satisfactory for various critical maneuvers and use cases [172]. Moreover, the literature studies have either used fewer datasets with minimum features (or) collected data from minimum sources to evaluate their decision models. Given the limitations of the datasets, it is difficult to gauge the effectiveness of the decision-making approaches described in the literature [143,144,146,173]. Furthermore, most of the relevant research literature did not focus on the ensemble concept and thus is unable to accommodate the newly arriving features or instant environmental changes. Moreover, the literature suffers from a lack of studying the efficiency, success, and failure rates of their proposed decision-making models. Finally, Section 4.3 discusses the impact of situation awareness on effective decision-making in AVs. The survey also discussed the major contributions and pitfalls associated with the four main areas focused on in this survey and also highlighted the limitation of existing models prevailing in the current research.

## Figures and Tables

**Table 1:** Summary of the panel ideas and issues of AI/ML and information fusion.

	SDF Problem	Use of ML	Challenges of ML	New Research
ABdelzaher	Physics-based and human-driven IF	Big data analysis if edge sensing	Unlabeled data	Coordination of DL through multiple AI/ML networks
Basch	User augmentation	High-dimensional learning	Heterogeneous analysis	Model based methods to address the unknown
Baines	Contextual support	Interpretable analysis	Determining various users	Explainable results
Chong	Data Association	Training from data	Relevant Models	Context-based AI
Koch	Perceiving and action	Data processing for object assessment	Combining data and models for situation assessment	Need common terms for ethical, social and usable deployment.
Leung	Image fusion	Change Detection	Real-time labeling	Joint multimodal image data fusion
Pham	Multi domain coordination	Rapidly learn, adapt, and reason to act.	Interface in sparse and congested areas	Learning in the edge

**Table 2:** Comparison of the different sensory devices.

Type	Advantages	Disadvantages	Max Working Distance
MMW-RADAR	1. Long working distance 2. Available for radial velocity 3. Applicable for all weather conditions	1. Not suitable for static objects 2. Frequent false alarm generation	5–200 m
Camera	1. Excellent discernibility 2. Available lateral velocity 3. Available for color distribution	1. Heavy calculation 2. Light interference 3. Weather susceptible	250 m (depending on the lens)
LiDAR	1. Wide field of view 2. High range resolution 3. High angle resolution	1. Insufferable for bad weather 2. High price	200 m
Ultrasonic	1. Inexpensive	1. Low resolution 2. Inapplicable for high speed	2 m
DSRC	1. Applicable for high speed (up to 150 km/h) 2. Relatively matured technology 3. Low latency (0.2 ms)	1. Low data rate 2. Small coverage	300–100 m
LTE-V2X	1. Long working distance 2. Relatively high data transmission rate (Up to 300 mbps)	1. High latency in long distances (>1 s) 2. Inapplicable for time-critical events	Up to 2 km
5G-V2X	1. Ultra-high data transmission rate 2. Low latency (<80 ms) 3. High bandwidth 4. Applicable for high speed (up to 500 km/h)	1. Immature application	100–300 m

**Table 3:** Task analysis based on sensor fusion.

Scenario	Specific Task	Ref	Sensor Types
Perception of Moving Objects in Traffic Environment (Including pedestrians, bicycles, vehicles, etc.)	Pedestrians	[56,80]	L
		[81]	RL
		[57,82]	CL
		[83]	RC
	Vehicle	[62,83,84,85,86,87]	RC
		[88,89]	CL
		[90]	RCLUV
		[91,92]	GI
		[93]	GL
		[94]	VG
		[95]	LV
		[81]	RL
		[96]	CLGI
		[97]	RL
	Pedestrian and Vehicle	[98]	R
		[88,99,100,101]	RC
		[102]	RCLGI
		[103]	LV
		[47]	CL
		[51]	CL
	Lane detection, Obstacle Detection, and Path Planning	[104]	RCLG
		[105]	RCL
		[58,82]	CL
		[95]	VG
		[77]	CG
		[106]	L
Reconstruction and Visualization of the Front Area	Safety Zone Division	[107]	RC
		[108]	RC
	Motion Analysis	[96]	CLGI
		[82]	CL
	Visualization	[80]	L
		[44]	RC
		[69]	CL
Safety Zone Construction and Collision Warning	Obstacle Avoidance	[109]	CL
		[87]	RC
		[109]	C
		[94]	VG
	Safety Zone Division	[107,110]	RC
		[43]	LC
Multisensory Calibration and Data Fusion Platform	Data Fusion Platform	[76,111,112]	N/A
		[112]	RCL
		[69]	CLGI
	Multi sensor Calibration	[113,114]	RC
		[115,116,117]	CL
Object Characteristics	Driver Behaviour	[51]	CL
Occupancy Probability	Parking Space	[68]	CL

**Table 4:** Frequently used datasets for AVs.

Dataset	Year	Hours	Traffic Scenario	Diversity
KITTI [118]	2012	1.5	Urban, Suburban, Highway	-
Waymo [119]	2019	6.4	Urban, Suburban	Locations
nuScenes [120]	2019	5.5	Urban, Suburban, Highway	Locations, Weather
ApolloScape [121]	2018	2	Urban, Suburban, Highway	Weather, Locations
PandaSet [122]	2021	-	Urban	Locations
EU Long-term [123]	2020	1	Urban, Suburban	Seasons
Brno Urban [124]	2020	10	Urban, Highway	Weather
A * 3D [125]	2020	55	Urban	Weather
RELLIS [126]	2021	-	Suburban	-
Cirrus [127]	2021	-	Urban	-
HUAWEI ONCE [128]	2021	144	Urban, Suburban	Weather, Locations

**Table 5:** Chronological summary of various fusion methods (ML—machine learning, MM—mathematical model).

Citation	Model	Advantages	Disadvantages	Drawbacks Rectified from Previous Study (?)
[120]	MM	Use of large 3D dataset for evaluation	Have not discussed image point level semantic labels.	✓
[49]	MM	Effectively handled 3D image data using CARLA	Unexploited to evaluate multisensory data	✓
[50]	MM	Evaluated their model using heterogeneous dataset.	Missed testing in real-time robotic environment.	✓
[129]	ML	Better in handling larger datasets in real-time scenarios.	Not expected level of accuracy (74%)	✓
[130]	ML	Enhanced accuracy (84%)	Can use advanced Fuzzy concepts optimize their model	✓
[131]	ML	An effective optimization method for heterogeneous data	Difficulty in obtaining proper metrics.	✓
[132]	ML	Involved more metrics to enhance the accuracy	Missed collecting data from different sensor points	✓
[68]	ML	Uses different data points collected using magnetic sensors	Complicated fuzzy rules	✓
[133]	ML	More accuracy	Evaluation done with minimum dataset.	✓
[66]	ML	Outperforms existing LiDAR and image based fusion models	Consumes more optimization time	✓
[97]	ML	CNN framework improves optimization time of fusion	Minimum classes.	✓
[67]	ML	Multiple classification	Needs changes in network topologies and training techniques	✓
[51]	ML	Eliminates the need to design and train separate network blocks	Missing complex multimodal context	✓
[134]	ML	Uses multiple inputs	Did not use structural restrictions.	✓
[69]	ML	Used structured CNN architecture to fuse data	Lacks optimization problems.	✓
[49]	ML	Better optimization of parameters using advanced statistics	Needs more real-world evaluation	✓
[135]	ML	Advanced image transformation using real time data.	The model misses tackling data missed from defective sensors.	✓

**Table 6:** Behavior of AVs over the distance covered.

Manufacturer	2015	2016	2017
Google/Waymo	1244	5128	5596
Nissan	14	146	208
Delpi	41	17.5	22.4
Mercedes Benz	1.5	2	4.5

**Table 7:** Chronological summary of the decision-making for Lane Change (LC) and Collision Avoidance (CA).

Citation	Event	Advantages	Disadvantages	Drawbacks Rectified from Previous Study (?)
[141]	LC	Instant decisions making (Intelligent agent)	More time-consuming to train new models	✓
[142]	LC	Minimizes training time	Missing detailed analysis on various lane changing scenarios	✓
[143]	LC	Considers all lane changing possibilities (85%) accuracy	Used limited datasets to evaluate the model	✓
[144]	LC	Optimal decision-making	Efficiency of model over many metrics not discussed properly.	✓
[145]	CA	Makes effective decision in limited visibility scenarios	Requires more detailed analysis	✓
[146]	CA	More decision rules	Evaluation done with minimum dataset	✓
[147]	CA	Effective decision-making with constrained dynamic optimization	Needs adaptive optimal mechanisms to handle dynamic scenarios	✓
[94]	CA	Innovative DSRC based model to avoid rear-end collision	Needs more real-time scenarios to evaluate the model	✓

**Table 8:** Chronological summary of decision-making for multiple roadside events (ME).

Citation	Event	Advantages	Disadvantages	Drawbacks Rectified from Previous Study (?)
[148]	ME	Frames multiple decision rules	Short response time, safety mechanism not included	✓
[149]	ME	Covers safety metrics. Uses various features for model evaluation	Uses minimum dataset for model evaluation	✓
[150]	ME	Model works for both AVs and CAVs	Difficulty in accessing real word CAV data	✓
[154]	ME	Use real-time data for evaluation	No full-fledged data for evaluation (partially observed)	✓
[151]	ME	Discuss various possibilities of pedestrian behavior	Needs more research in ideal communication method between AVs and pedestrians	✓
[152]	ME	Better optimized approach for overtaking and tailgating decisions	Fewer cases to evaluate the model	✓
[153]	ME	Model tested using real-time data.	Efficiency of the model not discussed.	✓

**Table 9:** Chronological summary of decision-making for platooning (PL) in AVs.

Citation	Event	Advantages	Disadvantages	Drawbacks Rectified from Previous Study (?)
[155]	PL	Simplifies the demand in resource poling.	Limited scenarios used for evaluation.	✓
[156]	PL	Uses more evaluating scenarios.	Cannot handle complex scenarios.	✓
[157]	PL	Handles larger datasets.	Time-consuming to optimize the model.	✓
[158]	PL	Good optimized model to handle spatial data.	Needs to consider mixed platooning issues.	✓
[159]	PL	Considers all platooning cases.	Latency issues affecting accuracy.	✓
[160]	PL	Can better handle emergency scenarios.	Needs valid proof to verify decision-making.	✓

**Table 10:** Summary of Decision-making in AV (LC—Lane Crossing, AC— Accidents, PC—Pedestrian Crossing, CC—Collision Conditions, and AI/ML—Artificial Intelligence/Machine Learning, MM—Mathematical Models.

Reference No.	Vehicle Type		Category		Events				Methodology	
	**AV**	**Manual**	**Survey**	**Model**	**LC**	**AC**	**PC**	**CC**	**AI/ML**	**MM**
[167]	✓	×	✓	×	✓	×	×	×	×	✓
[144]	✓	×	×	✓	✓	×	×	×	×	✓
[153]	✓	×	×	✓	✓	✓	×	×	✓	×
[143]	✓	×	×	✓	×	×	×	×	×	✓
[148]	✓	×	×	✓	✓	✓	×	×	×	✓
[146]	✓	×	×	✓	✓	✓	×	×	✓	✓
[152]	✓	×	×	✓	✓	✓	✓	×	×	✓
[149]	×	✓	✓	×	✓	✓	×	×	×	×
[154]	✓	×	×	✓	✓	×	×	×	×	✓
[151]	✓	×	×	✓	×	×	✓	×	✓	×
[150]	✓	×	×	✓	×	✓	×	×	✓	×
[160]	✓	×	×	✓	✓	✓	✓	×	×	✓
[147]	✓	×	×	✓	✓	✓	×	✓	×	✓

## Data Availability

This is an extensive survey, hence we don’t use or create any dataset for analysis.
